# Selective formation of tungsten nanowires

**DOI:** 10.1186/1556-276X-6-543

**Published:** 2011-10-04

**Authors:** Daniel CS Bien, Rahimah Mohd Saman, Siti Aishah Mohamad Badaruddin, Hing Wah Lee

**Affiliations:** 1 Nanoelectronics Cluster, MIMOS Berhad, Technology Park Malaysia, 57000 Kuala Lumpur, Malaysia

**Keywords:** tungsten, nanowires, nanostructures, self-aligned, chemical vapor deposition, selective deposition

## Abstract

We report on a process for fabricating self-aligned tungsten (W) nanowires with polycrystalline silicon core. Tungsten nanowires as thin as 10 nm were formed by utilizing polysilicon sidewall transfer technology followed by selective deposition of tungsten by chemical vapor deposition (CVD) using WF_6 _as the precursor. With selective CVD, the process is self-limiting whereby the tungsten formation is confined to the polysilicon regions; hence, the nanowires are formed without the need for lithography or for additional processing. The fabricated tungsten nanowires were observed to be perfectly aligned, showing 100% selectivity to polysilicon and can be made to be electrically isolated from one another. The electrical conductivity of the nanowires was characterized to determine the effect of its physical dimensions. The conductivity for the tungsten nanowires were found to be 40% higher when compared to doped polysilicon nanowires of similar dimensions.

## Background

One-dimensional nanostructured materials such as nanowires, nanorods, and nanotubes have been the focus of intensive research owing to their unique applications in mesoscopic physics and novel nanoscale devices. These nanostructures provide a good platform to investigate the electrical, thermal transport, and mechanical property dependence on dimensionality and size reduction.

Tungsten is a brittle refractory metal that crystallizes in body-centered cubic form. It has high tensile strength and good creep resistance. Due to its high stability, tungsten or tungsten oxide nanowires are promising candidates for a vast range of applications including, smart coatings [1], lithium-ion batteries catalysts [2], electrochromatic materials [3,4], and nanostructured sensors [5,6].

Nanowires are unique for sensing applications as they exhibit high sensitivity, long-term stability, and large surface to volume ratios. For sensing applications, tungsten or tungsten oxide nanowires, are known to have a high sensitivity for detecting gasses such as ammonia [7,8], nitrogen dioxide [9,10], hydrogen sulfide [11,12], hydrogen [6,13], pH [5], and etc. at low parts per million and even as low as parts per billion levels.

In semiconductor fabrication, there are various methods that can be used to fabricate patterned nanowires. However, organizing these nanowires into highly ordered arrays can be extremely challenging. Metallic nanowires can be produced with a combination of advanced lithography [14-16], metal etching, chemical mechanical planarization [17], and metal lift-off [18-20]. However, these techniques have limitations and are typically not cost-effective. Metal lift-off with sacrificial resist is a more common solution for producing nanostructures, but the process has resist-imposed limitations namely the thermal stability of the resist which prevents its use in a chemical vapor deposition (CVD) metal process. In this letter, the authors demonstrate a novel method for fabricating tungsten nanowires which allows full integration with standard CMOS fabrication process. The method utilizes selective deposition [21] as an alternative to the conventional growth of nanowires using metal catalyst to form the nanowire structures. The key feature of this method is the ability to selectively deposit and align tungsten nanowires on silicon or polysilicon lines utilizing selective CVD processing. The precursor used, tungsten hexafluoride (WF_6_), only reacts with silicon or polysilicon material but will not react with insulating material, such as silicon dioxide or silicon nitride. The method demonstrated here is cost-effective and circumvents the need for state-of-the-art equipments. There are also no lithographic limitations and the nanowires produced are of high resolution. The process is also self-limiting with good control of nanowire diameters that are less than 50 nm. Another advantage of this method is that the tungsten nanowires can be produced or synthesized at temperatures below 400°C without the need for metallic catalyst, which are commonly used in catalytic reaction synthesis with growth temperatures typically in a range of 700 to 1,000°C [9,22-25]. Hence, our proposed method allows the use of low-temperature substrates such as polymer or glass, which facilitates manufacturing flexibility and reduces costs.

## Method

**Figure 1:**
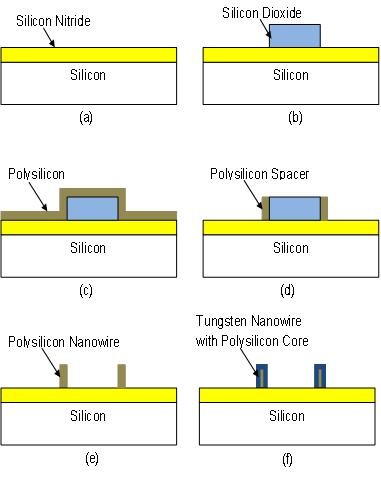
**Fabrication process flow to selectively form tungsten nanowires with polysilicon core**.

Figure 1 illustrates the fabrication process of the tungsten nanowires. All experiments were conducted on 200-mm-diameter silicon wafers. A 200-nm-thick silicon nitride (Si_3_N_4_) layer was deposited at 800°C by low-pressure chemical vapor deposition (Figure 1a) to serve as electrical isolation between the nanowires and silicon substrate. This was followed with a 100-nm-thick silicon dioxide (SiO_2_) layer deposited by plasma-enhanced chemical vapor deposition which was patterned into lines by standard photolithography and etched in CF_4 _and CHF_3 _plasma (Figure 1b). A 50-nm-thick undoped polysilicon film is then deposited by low-pressure chemical vapor deposition, PECVD (Figure 1c). Polysilicon spacers were produced by time-controlled etching of the polysilicon layer with HBr and CF_4 _plasma in a reactive ion-etching system (Figure 1d). The width of the spacers or would be final polysilicon nanowires is in correlation with the deposited thickness of the PECVD polysilicon film and dependent on the directionality of the plasma etch. The silicon dioxide layer was then selectively etched in buffered hydrofluoric acid (HF) resulting in 50-nm polysilicon nanowires formed on silicon nitride (Figure 1e). The etch rate of SiO_2 _in buffered HF was approximately 70 nm/min while the etch rate of Si_3_N_4 _in the same solution was approximately 2 nm/min, showing high selectivity. The fabricated polysilicon nanowires are comparable to that in literature from sidewall transfer technology [26-28]. Finally, the tungsten nanowires were formed by selectively depositing tungsten by CVD at 400°C onto the exposed polysilicon nanowires (Figure 1f). More information on the CVD conditions is detailed in the next section.

## Results and discussions

**Figure 2:**
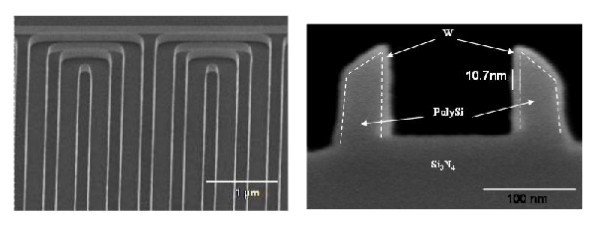
**SEM cross-sectional images**. (**a**) An array of tungsten nanowires; (**b**) magnified to depicting tungsten thickness of 10 nm.

The tungsten nanowires exhibited good adhesion to the polysilicon core and the underlying silicon nitride surface, where nanowire thickness down to 10 nm was achieved (Figure 2). The tungsten deposition is confined to the polysilicon regions; hence, the nanowires are electrically isolated from one another without requiring processing such as etching. There is also no limit to the length of the nanowire that can be produced with this method.

The selective tungsten deposition chemistry confining the tungsten to only polysilicon regions, is based on the reduction of the tungsten hexafluoride (WF_6_) precursor by the exposed polysilicon as described in Eq. 1; hence producing the selectively formed tungsten nanowires

(1) $$\text{2W}{\text{F}}_{\text{6}}\left(g\right)+\text{3Si}\left(s\right)®;\text{2W}\left(s\right)+\text{3Si}{\text{F}}_{\text{4}}\left(g\right)$$

Tungsten deposition was performed at 400°C with 10 sccm of WF_6 _precursor with 500 sccm of Argon carrier gas for 5 min, yielding a layer of approximately 10 nm thick with a resistivity of approximately 13 μΩcm. The sheet resistance of a blanket 10-nm-tungsten layer was measured using a four-point-probe. The by-product from this reaction is silicon tetrafluoride, which is nonreactive with semiconducting material. Argon was used as the carrier gas to aid the removal of these by-products from the wafer surface, thus reducing deposited layer resistivity. The achieved resistivity compares well to published resistivity of 20-μΩcm for a 100-nm-thick tungsten film selectively deposited on bulk silicon [29]. It is proposed that in the future, the underlying polysilicon can be doped to further reduce the resistivity of the nanowire. The tungsten deposition temperature has an impact to the selectivity of the process, where at 450°C nucleation was observed on the silicon nitride surface. When the deposition temperature is at 500°C, selectivity was lost in which tungsten was deposited across the whole substrate on both polysilicon and silicon nitride surfaces.

**Figure 3:**
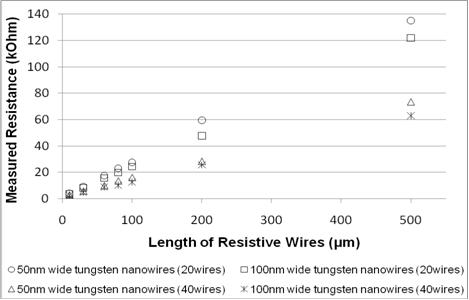
**Electrical resistances of fabricated tungsten nanowires with varied dimensions**.

**Figure 4:**
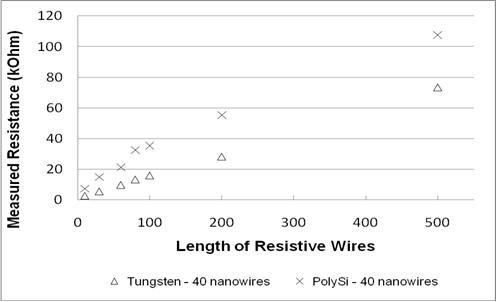
**Measured resistance, comparing tungsten nanowires with doped polysilicon nanowires with similar dimensions**. Polysilicon width of 50 nm.

Electrical resistances of the fabricated tungsten nanowires were characterized to determine the effect of nanowire length and width as shown in Figure 3. The lengths of the measured nanowires were varied from 20 to 500 μm, and it was observed that the resistance increases almost linearly with length. Inversely, the measured resistance increases by approximately 10% when the width of the core is reduce from 100 to 50 nm. As we double the number of wires from 20 to 40, the measured resistance reduces by 40% due to the increase in the total measured surface area. Comparing these results to those of doped polysilicon nanowires with similar dimensions, the electrical resistance measured for tungsten nanowires were found to be at least 40% lower (Figure 4).

## Conclusions

In summary, we have demonstrated a self-align process to produce highly ordered arrays of lateral tungsten nanowires utilizing a combination of sidewall transfer technology and selective tungsten CVD. All nanowires were produced with a polysilicon core. Overall, the fabrication process does not require sub-micron lithographic techniques, metal catalyst, metal lift-off, extensive etching, or polishing. A 5-min tungsten deposition at 400°C is sufficient to produce 10-nm-thick tungsten nanowire with high selectivity and good adhesion to the underlying layers. The measured electrical resistances for these wires were found to increase almost linearly with length of the nanowires and are 40% more conductive when compared to doped polysilicon nanowires of the same dimensions. We believe the process is easily scalable to assemble tungsten nanowires with sub-50-nm polysilicon core.

## Competing interests

The authors declare that they have no competing interests.

## Authors' contributions

DCSB coordinated the research work, conceived the fabrication process flow and conducted the electrical characterization for the nanowires. RMS conducted the selective tungsten deposition experiments. SAMB fabricated the polysilicon nanostructures and HWL designed the nano-resistor layout and participated in the electrical characterization of the nanowires. All authors read and approved the final manuscript.
